# Menstrual hygiene management practice and associated factors among high school and preparatory school adolescent students in Debre Markos town, Northwest, Ethiopia: a mixed-method study

**DOI:** 10.1186/s12905-024-03265-y

**Published:** 2024-07-25

**Authors:** Yichalem Worku, Getachew Mullu Kassa, Bekele Mekonen, Melaku Desta, Keralem Anteneh Bishaw, Mihretie Gedfaw, Eyerus Tesfaw, Genet Degu, Aster Shiferaw

**Affiliations:** 1 Department of Midwifery, College of Medicine and Health Sciences, Debre-Markos University, Debre-Markos, Ethiopia; 2 School of Nursing, Johns Hopkins University, Baltimore, MD USA; 3 Department of English Language and Literature, College of Social Sciences, Debre-Markos University, Debre-Markos, Ethiopia; 4 Department of Nursing, College of Medicine and Health Sciences, Debre-Markos University, Debre-Markos, Ethiopia; 5 Department of Public Health, College of Medicine and Health Sciences, Injibara University, Injibara, Ethiopia

**Keywords:** Menstruation, Adolescent girls, Hygienic management, Mixed study, Ethiopia

## Abstract

**Background:**

Despite being a normal occurrence, menstruation requires hygienic care and is associated with a number of myths and wrongdoings. Menstrual hygiene issues have been linked to major health issues, such as urinary tract and reproductive tract infections. Consequently, the purpose of this study was to evaluate the management of menstrual hygiene and related aspects among teenage students in Debre Markos town, North West, Ethiopia.

**Methods:**

From March 15 to April 15, 2019, teenage pupils in Debre Markos town participated in a cross-sectional mixed study. An in-depth interview and a self-administered structured questionnaire were used to gather data. Quantitative information was imported into Epi Data and then exported to SPSS for examination. A 95% Confidence Interval of *p*-value ≤ 0.05 was used to declare significance. The method of thematic content analysis was used to examine the qualitative data.

**Result:**

This study comprised 531 individuals in total, with a 96.2% response rate. Approximately 260 adolescent females (49%, 95% CI: 39.2, 59.2) had good management practices for menstrual hygiene. Girls whose mothers were private employees (AOR: 0.3, 95% CI: 0.09, 0.99), self-employed (AOR: 0.52, 95% CI: 0.28, 0.98) and housewives (AOR: 0.53, 95% CI: 0.29, 0.98), and parent-adolescent discussions about menstruation (AOR: 1.62, 95% CI: 1.40, 3.34) were significantly associated with good menstrual hygiene management. Adolescence aged 17 years or older (AOR: 2.13, 95% CI: 1.32, 3.44) were found to have good knowledge regarding menstrual hygiene (AOR: 1.59, 95% CI: 1.43, 2.94). The qualitative study found that ignorance, an unfavorable school climate, and cultural and economic factors were the main causes of teenagers’ poor menstrual hygiene.

**Conclusion:**

Nearly half of adolescent girls had good menstrual hygiene practice. Menstrual hygiene practice was associated with adolescent age, knowledge of menstruation, maternal occupational and discussion with parents. Girls had difficulty to manage menstrual hygiene due to poor knowledge, unsafe school environment, and cultural barriers. Thus, school-based programs aimed at improving knowledge towards menstrual hygiene management are needed.

**Supplementary Information:**

The online version contains supplementary material available at 10.1186/s12905-024-03265-y.

## Introduction

Between the ages of 10 and 19, adolescence is a unique stage of life marked by profound changes in one’s physical, mental, emotional, and social makeup. It is also the time when development determines whether one will have a healthy or unhealthy adult life [1]. One of the most significant physiological changes that teenagers experience—and one that may have long-term effects on their lives—is the commencement of menstruation. The majority of these women and girls have two to seven days of menstruation every month [2]. It is estimated that a woman will experience menstruation for around 43,200 h of her life, or roughly 5 years of bleeding. Menstrual cycles typically last between 21 and 35 days, with an average of 28 days [3].

Although the menstrual cycle is a physiological process, it nevertheless requires sanitary care and is associated with several myths and habits that may have an adverse effect on women’s health. Menstrual hygiene issues have been linked to major health issues, such as urinary tract and reproductive tract infections. Girls go through a range of emotions during their first menstrual cycle, including worry, guilt, dread, and embarrassment because they didn’t know anything about the event beforehand [4–8].

During the first few days of severe bleeding during menstruation, women must replace their sanitary napkins. They also require a private space to change and discard used materials, as well as access to clean water and soap for washing. They might need to change their sanitary napkins every three to four hours during the heaviest days of their menstruation [5, 9, 10]. Most girls change using cotton pads rather than sanitary napkins, which need to be washed and reused multiple times. Much of the disease and infection linked to female reproductive health is caused by a culture of shame and embarrassment [3, 11–13].

Poor menstrual hygiene management (MHM) is linked to a lack of hygienic, functional, private, gender-specific water, sanitation, and hygiene (WASH) facilities in school settings as well as limited access to sanitary supplies [4]. Absenteeism can lead to subpar academic performance, dropout rates, and lower educational attainment, all of which have detrimental long-term effects on gender equality as well as the economy and health [2, 5–7, 14]. Because they feel ashamed, worry about having noticeable stains on their clothes, lack a private space to handle their period, or are afraid of what will happen to their body when they reach menarche, many school-age girls skip school during their periods, which can be frightening, stressful, and confusing [15].

Due to their monthly periods, females may miss up to 20% of school worldwide, and 10% will drop out. Up to 95% of girls in the Middle East and Asia claim that their menstruation is the reason they are missing school [2, 16, 17]. About 40% of students in Delhi, India [18] and 95% of girls in Ghana miss school due to menses, 86% girls in Garissa in Kenya miss one or more days of school, 7% of girls abstain from school on heavy days in Malawi [19] and 54.5% girls in Ethiopia have been absent from school during their menstruation period [2, 20, 21].

Schools are crucial venues for enhancing MHM, because they have significant influence on the vast majority of teenagers that can be reached by educational interventions aimed at enhancing MHM behaviors and understanding while addressing harmful myths, cultural taboos, and stigma. The management of menstrual hygiene has a direct impact on the environment, education, development, and physical and mental health. It is also crucial to achieving the sustainable development goals (SDGs). In particular, Sustainable Development Goals (SDGs) 3 (healthy physical and mental-social development for women and girls), 4 (quality education for all girls), 5 (equality and empowerment of women and girls), 6 (water and sanitation), and 12 (responsible consumption and production for the environment) [22].

According to a research conducted in Mahalmeda, Ethiopia, just 9.1% of the girls had poor menstrual hygiene; of those, 26% of schoolgirls created their own sanitary pads and 29.7% of them reused them [21]. Similar study done in Northwest Ethiopia showed that 70.2% of the girls practiced poor menstrual hygiene [23], and 32% of adolescent girls practiced poor menstrual hygiene [37].

One of the biggest issues adolescent girls in Ethiopia deal with in school is managing their menstrual hygiene. The main causes of this are cultural taboos, a lack of parental advice and information about MHM, inadequate facilities and bad facility management, a lack of support, and a lack of knowledge. A Southern Ethiopian study revealed that the school environment was unsuitable for MHM, with 90% of the schools lacking a water supply and separate restrooms for boys and girls [24].

However, this issue receives little attention, and there are few studies conducted in Ethiopia, especially in the study region, on menstruation, hygienic management, and its impact on girls’ education. The majority of research was also done in a quantitative manner. Up until our search, no study had been conducted in our nation that used a standard instrument to quantify social support. Thus, the purpose of this study was to evaluate the management of menstrual hygiene and related characteristics among adolescents enrolled in high school and preparatory school in Debre Markos Town, East Gojjam Zone, Northwest Ethiopia.

## Methods

### Study design, setting and period

Using a cross-sectional study design, a convergence triangulation mixed method was carried out from March 15 to April 15, 2019. In Debre Markos Town, East Gojjam Zone, Ethiopia, high school and preparatory teenage students participated in the study. The town of Debre Markos is the administrative center of the East Gojjam region. The settlement is located 265 km from Bahir Dar city and 300 km northwest of Addis Ababa, the capital of Ethiopia. The city is home to three high schools, two preparatory schools, and twelve junior schools (eight public and four private). There are 2,273 female students and 1,995 male students enrolled in grades 9 and 10 of the 4,268 adolescents enrolled in the 2018–19 academic year. Additionally, there are 2894 adolescents enrolled in grades 11 and 12. In DM/general, there were 20 sections of grade 9 and 20 sections of grade 10; in Menkorer, there were 10 sections of grade 9 and 7 sections of grade 10; in Ethio-Japan, there were 8 sections of grade 11 and 7 sections of grade 12; and in Mesenado schools, there were 14 sections of grade 11 and 12 sections of grade 12 [25].

### Population and eligibility criteria

The source populations were all female teenage students enrolled in grades 9–12 at Debre Markos Town High School and Prep School for the 2018–19 academic year. The study population consisted of all regularly selected, randomly selected, female teenage students (ages 10 to 19) enrolled in grades 9 through 12 at Debre Markos Town High School and Prep School in 2018–19 and attending classes during the data collection period. Key informants for the qualitative study included parents, instructors, school administrators, students, and the manager of the health office. All female adolescent students in grades 9 through 12 who were enrolled in a regular program and aged 10 to 19 met the qualifying requirements for the quantitative portion of the study, while those with additional knowledge regarding menstruation and adolescents were included in the qualitative portion. Students who refused to participate in interviews when they were menstruating, however, were not included in the study.

### Sample size and sampling procedures

Using Epi-Info software, the sample size was determined by applying a single population proportion formula for the magnitude of MHM and characteristics that have a significant association with MHM. The sampling strategy took into account a 95% confidence interval, 5% error margin, 80% power, and 10% non-response rate. The sample size was determined using data from earlier Ethiopian research projects [23, 24]. Finally, the highest sample size was selected from a study conducted in Adama town [24]. As a result, 552 teenage girls made up the quantitative portion of this research, and 12 key informants were chosen for the qualitative portion of the study using the purposive sampling technique.

**Fig. 1:**
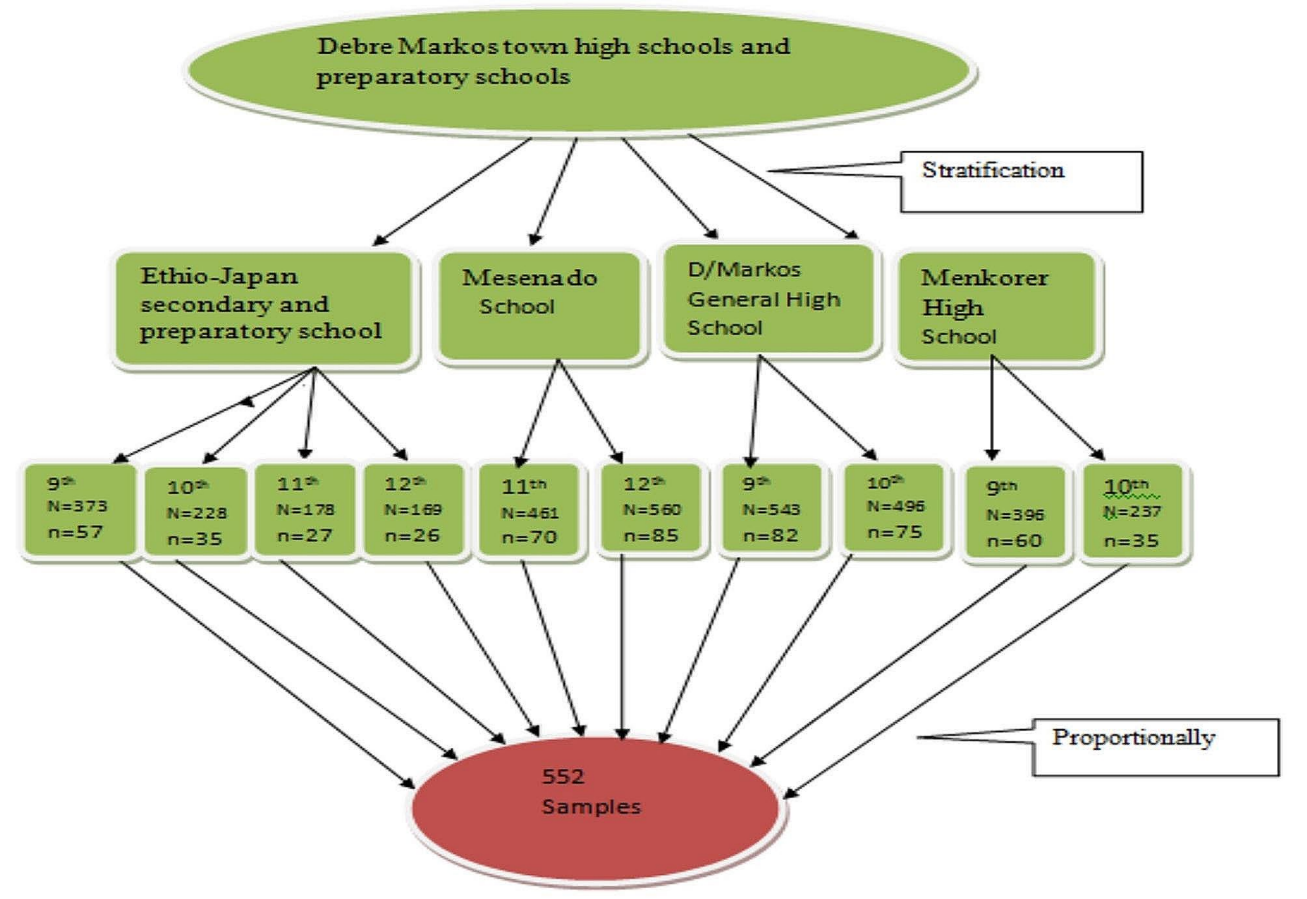
Graphical presentation of sampling procedure of menstrual hygiene practice among high school and preparatory school adolescent students, Debre Markos town, Ethiopia, 2019

During the research period, Debre Markos town had two preparatory schools (Mesenado and Ethio-Japan, which has grades 9 through 12), and three high schools (Ethio-Japan, Menkorer, and Debre Markos General). This survey encompassed all high schools and preparatory schools. Using the proportionate to sample size allocation approach, the sample size was distributed among all high schools and preparatory schools. The school register provided the sampling frame for each school. Then, systematic random sampling method was used to randomly select study participants from each school (Fig. 1) Purposive sampling was used in the qualitative study to select participants for the in-depth interview based on their working location and occupation. The principal investigator established contact and arrangements with the school directors and families in order to address the study participants.

### Variables and measurements

The study’s dependent variable was the management of menstrual hygiene practices. Three categories comprised the independent variables: menstrual hygiene (accessibility of social support during menstruation, type and availability of sanitary materials, sanitary material washing and drying condition, knowledge of menstrual hygiene, and open communication about menstruation with parents), school environment (water and latrine availability, school comfort or private place to manage menses and waste disposal), and sociodemographic and economic (age, residency, mother and father’s educational and occupational status, family size, pocket money, and monthly income).

The term “menstrual hygiene management practice” refers to the following: washing genitalia and reusable pads at a sufficient frequency (two times or more per day); bathing the body in an exceptional manner; adequate disposal (other than in an open area); and privacy (not being watched by others or in a comfortable place). Women and adolescent girls should use clean absorbents (disposable sanitary pads or reusable pads that have been cleaned and dried in the sun) [23, 24, 26, 27]. If respondents had a score of five or higher on the seven practice-related items, they were categorized as having good menstrual hygiene management practices; if they received a score of less than five, they were classed as having poor menstrual hygiene management practices. An extra 10-item test was employed to assess participants’ knowledge of teenage girls. If a respondent scored eight or more on the ten knowledge-related items, they were classed as having strong knowledge of menstrual hygiene; if they scored less than eight, they were classified as having inadequate knowledge [23, 24]. Additionally, a range of 3 to 14 was observed in the results of the Oslo Social Support Scale, which was used to quantify social support. Nine to eleven was categorized as “moderate social support,” twelve to fourteen as “strong social support,” and three to eight as “poor social support.” [28].

### Data collection procedure and quality control

To assess the management of menstrual hygiene and related difficulties, self-administering structured questionnaires were used. The data collection instrument comprised five elements, which were associated with menstruation hygiene, social support, school environmental characteristics, and sociodemographics. (31). The data collection tool was first developed in English, translated into Amharic, and then back into English to guarantee consistency. A random sample of female students was invited to provide data after the technique was explained to teachers and research participants.

In-depth interviews with interview guides were utilized to gather data for the qualitative study using semi-structured open-ended interview questions. Further in-depth questions were asked in response to the participants’ answers. Although the interview guides were written in English, the participants spoke Amharic throughout the interviews. The interview guides contained questions about sociodemographic characteristics as well as those relating to the environment, education, families, and cultures. During the in-person interview, the investigator had one-on-one conversations with each respondent. During the interview, field notes were taken. Data were recorded using a recorder.

Female students in one high school and one preparatory in Finote-Selam town were given a pretest that made up 5% (28) of the total sample size. The internal consistency test using Cronbach alpha (0.8) was employed to evaluate the reliability of the tool. Two days of training were provided for supervisors and data collectors. The accuracy and consistency of the data were checked daily. Trust was established between the interviewer and respondents in order to collect qualitative data. The suitability and clarity of the interview rules were evaluated by senior professionals. During the data collection phase, notes were taken and interviews were held.

### Data processing and analysis

The quantitative data was entered, verified, and cleaned using Epi Data version 3.1 software. It was then exported to the version 25 of the Statistical Package for Social Science (SPSS) software for more analysis. Data cleaning was done to confirm for consistency and frequencies. The model’s fitness was evaluated using Hosmer and Lemeshow goodness of fit test statistics, and the findings showed that the model was fitted with a *p*-value of 0.6. Variables having a *p* value of ≤ 0.2 in bi-variable logistic regression were included in multi-variable logistic regression, and significant factors were identified at 95%CI at *p*-value of < 0.05. The data for the qualitative study were transcribed and scrutinized after every interview. The field note was amended again based on the findings, and the recordings were listened to multiple times in order to properly understand the concepts of each participant’s response. In the data presentation, pertinent quotes were arranged in a sequential fashion to support the main numeric results.

### Ethical consideration

Ethical clearance was granted by Debre Markos University and the College of Health Sciences Ethics Committee, with reference number HSC/989/16/18. Authorization was ensured by obtaining official letters from the College of Health Science and forwarding them to selected educational institutions. The confidentiality of the study was maintained at all times. Researchers using both quantitative and qualitative methods included willing participants in their studies. The girls under the age of eighteen were admitted with permission from their families or guardians.

## Results

### Socio-demographic characteristics

A total of 531 respondents—or 96.2% of the sample—completed the interview out of 552 respondents. Participants ranged in age from 13 to 19, with a mean age of 17.18 and a standard deviation (SD) of ± 1.21. With a standard deviation of ± 3.64, the menarche age was 14.4. Every respondent was of Amhara ethnicity, and 69.1% of research participants were between the ages of 17 and 19. Of the participants, around two-thirds (78.3%) lived in metropolitan regions, and 38.8% were in grade nine. Of the respondents, 124 fathers (30.9%) had only completed elementary school, while 75% of the mothers were illiterate. Once more, the fathers of respondents made up 62.1% of the workforce and worked for themselves. And 321 (60.5%) did not consistently receive pocket money from their household. (Table 1).

**Table 1:** Socio-demographic characteristics of high school and preparatory school adolescent students, Debre Markos town, Ethiopia, 2019

Variables	Frequency	Percent
**Age group**		
13–16 years	164	30.9
17–19 years	367	69.1
**Age at menarche**		
< 12 years	65	12.5
13–15 years	369	69.5
> 16 years	97	18.3
**Grade level**		
9th	206	38.8
10th	125	23.5
11th	89	16.8
12th	111	20.9
**Residence**		
Urban	416	78.3
Rural	115	21.7
**Religion**		
Orthodox	515	97
Muslim	8	1.5
Others *	8	1.5
**Educational status of the father**		
Can’t read and write	135	25.5
Primary	164	30.9
Secondary	98	18.5
College level and above	133	25.1
**Educational status of the mother**		
Can’t read and write	199	37.5
Primary	133	25
Secondary	103	19.4
College level and above	96	18.1
**Occupational status of the father**		
Government Employee	124	23.4
Private Employee	45	8.5
Self-Employee	330	62.1
Farmer	29	5.5
Merchant	3	0.6
**Occupational status of the mother**		
Government Employee	65	12.3
Private Employee	15	2.8
Self-Employee	152	28.8
House wife	292	55.3
Merchant and students	4	0.8
**Income (in Ethiopian birr)**		
Below 500 birr	28	6.1
501–1500 birr	71	15.5
Above 1500 birr	358	78.3
**Regular pocket money**		
Yes	210	39.5
No	321	60.5
**Family size**		
≤ 5	271	52.8
≥ 6	242	47.2

*catholic, protestant

### Environmental related characteristics

About 15.5% of participants reported that there were no functional facilities at their school, whereas the majority of participants (62.5%) claimed that the restrooms were open. A total of 178 respondents, or 35.5%, reported that their school lacked water service. Only 105 respondents (19.4%) said their school provided menstruation counseling, whilst 63 respondents (11.9%) said the institution provided sanitary items. (Table 2)

**Table 2:** Environmental related characteristic among high school and preparatory school adolescent students, Debre Markos town, Ethiopia, 2019

Variables	Frequency	Percent
School have functional water source		
Yes	352	66.5
No	178	35.5
School have functional toilet facility		
Yes	450	84.7
No	81	15.5
Females and males toilets in the opposite directions		
Yes	422	79.5
No	109	20.5
Females’ toilets kept locked inside		
Yes	199	37.5
No	332	62.5
School giving guidance and counseling service on menstrual hygiene management?		
Yes	105	19.4
No	428	80.6
Any organization who gives sanitary materials in school		
Yes	63	11.9
No	468	88.1

### Social support related characteristics of respondents

**Fig. 2:**
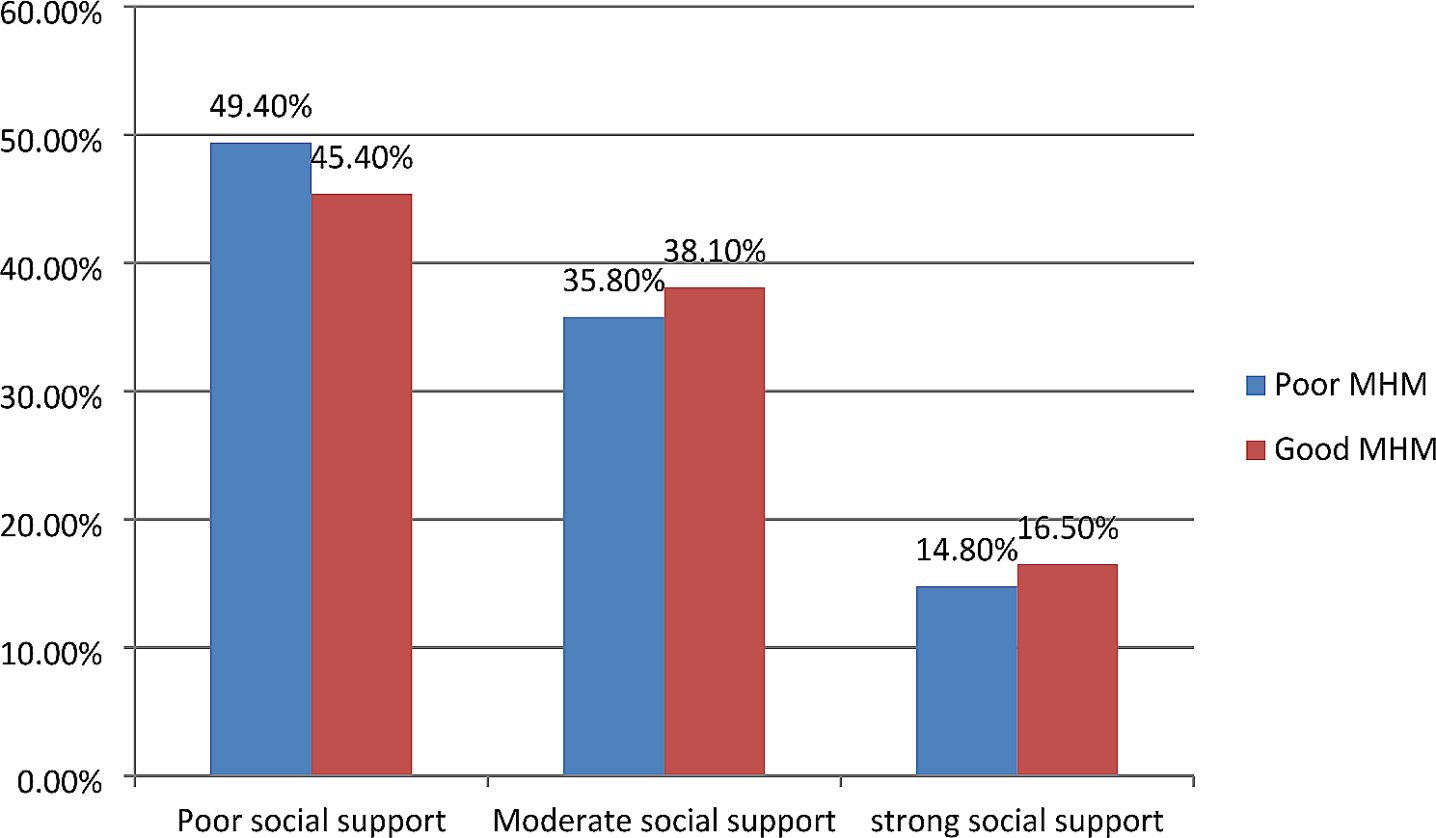
Social support related characteristics of respondents among high school and preparatory school adolescent students, Debre Markos town, Ethiopia, 2019

Just 11.7% of teenage females said that when they needed help with menstruation, they had no trouble approaching their neighbors for it. Table 3 demonstrates that fewer than one-third (21.5%) of the girls had more than five people within easy reach who could provide assistance in an emergency. Overall, more than half of the participants (47.5%) said they had insufficient social support, which was followed by moderate (36.7%) and strong (15.6%) social support. (Fig. 2)

**Table 3:** Social support related characteristics of respondents among high school and preparatory school adolescent students, Debre Markos town, Ethiopia, 2019

Variables	Frequency	Percent
How easy to get help from neighbours if it needed		
very difficult	86	16.2
Difficult	132	24.9
Possible	110	28.2
Easy	101	19
Very easy	62	11.7
How many people are so close to you that you can count on them if you have serious problems		
None	53	10
1-2	144	46
3-5	120	22.6
> 5	114	21.5
How much concern people in what you are doing		
No	46	8.7
Little	91	17.1
Uncertain	139	26.2
Some	125	23.5
A lot	130	24.5
Total	531	100%

### Knowledge towards menstrual hygiene

The majority (87.9%) said they had heard about menstruation before to starting their first period. In 21.5% of the cases involving teenage girls, mothers gave information about menstruation prior to menarche, but in 36.9% of the cases, peers did so. Similarly, according to 78% of participants, the menstrual cycle usually lasts between two and seven days. 69% (69.1%) of teenage females knew enough about menstrual hygiene to be comfortable with it. (Table 4).

**Table 4:** Knowledge towards menstrual hygiene among high school and preparatory school adolescent students, Debre Markos town, Ethiopia, 2019

Variables	Frequency	Percent
Heard about menstruation before menarche		
Yes	467	87.9
No	64	12.1
Source of menstrua information before menarche		
Mother	114	21.5
Teacher	152	28.6
Peers	196	36.9
Mass media	52	9.8
Books/other family members/neighbor/books	16	3
What is the cause of menstruation?		
Physiological process	455	85.7
Is caused by a sin	18	3.4
Is curse of God	36	6.8
Is caused by a disease	5	0.9
I don‘t know	17	3.2
Which organ the menstrual blood come from?		
Vagina	44	0.3
Urinary bladder	28	4.7
Uterus	345	65
I don‘t know	117	22
How long is normal menstrual bleeding duration?		
< 2 Days	36	6.8
2–7 Days	414	78
> 7 Days	30	5.6
Don’t know	51	9.6
What is the normal duration of menstrual cycle?		
< 20 Days	59	11.1
20–35 Days	347	65.3
> 35 Days	31	5.8
When do you think normal girl starts menses		
Don’t know	94	17.7
10–19 years	499	94
< 10 or > 19 years	32	6
Do you think menstruation is a secret issue?		
Yes	96	18.1
No	435	81.9
Know sanitary pads in the market?		
Yes	491	92.5
No	40	75
Poor menstrual hygiene predisposes to infection		
Yes	484	91.1
No	47	8.9
Menstrual hygiene has a contribution in prevention of menstrual pain.		
Yes	402	75.7
No	129	24.3
summary index for knowledge towards menstrual hygiene		
Good knowledge	367	69.1
Poor knowledge	164	30.9

*multiple responses were possible

### Menstrual hygiene management practice

Two hundred sixty adolescent girls (49%, 95% CI: 39.2, 59.2) reported using good management techniques for menstrual hygiene. Among the respondents, 81.6% reported using disposable sanitary pads, and 87.9% reported using sanitary items during their menstrual cycle. Of those who were menstrual, 64 people (12.1%) did not use sanitary napkins. The biggest obstacle to using sanitary products was their high price (33,55%), which was followed by people’s lack of knowledge about them. fifteen, or 25% (Table 5).

**Table 5:** Menstrual hygiene management among high school and preparatory school adolescent students, Debre Markos town, Ethiopia, 2019

Variables	Frequency	Percent
Use sanitary materials during menstruation		
Yes	467	87.9
No	64	12.1
Used sanitary materials during menstruation		
Disposable sanitary pads	381	81.6
Disposable piece of rags	30	6.4
Reusable sanitary pads	49	10.5
Underwear	6	1.3
Washing of genitalia during menstruation per day		
< 2 times	100	20.2
2 or more times	392	79.7
Bath during menstruation		
Yes	376	70.8
No	155	29.2
Dispose of menstrual materials after use		
Open field	15	2.8
Latrine	362	68.7
By paper and put bin	147	27.9
Others*	3	0.6
Storage of new and/or reusable absorbent		
Drawers	111	20.9
Dress cabinet	161	30.4
Bathrooms	135	25.5
Store with routine cloth	82	15.5
Others**	41	7.7
Places where reusable sanitary pads put after washing for drying		
in the sunlight outside	105	19.8
in the shade inside	410	77.4
others***	15	2.8
School comfortable to keep hygiene during menstruation		
Yes	173	32.6
No	358	67.4
Summery index for menstrual hygiene practice		
Poor practice	271	51
Good practice	260	49

*reuse, not use and burning. ** Alone, my bag, drawer and private place

*** Private place, bath room, firing, disposing and not reused

### Factors associated with menstrual hygiene management

The age of the respondent (*p*-value = 0.007), the respondents’ knowledge of menstruation (*p*-value = 0.007), the open discussion of menstruation issues with parents (*p*-value = 0.004), the mother’s occupation as a housewife (*p*-value = 0.024), and the mother’s occupation as a private employee (*p*-value = 0.049) were the factors that showed a significant correlation with practice, according to the bi-variable analysis of 14 variables. In order to load variables into the multivariate logistic regression model, *p*-values of less than 0.2 were required.

According to the results of the multi-variable logistic regression analysis, teenage girls older than 17 had a twofold higher likelihood (AOR: 2.13, 95% CI: 1.32, 3.44) of practicing good menstrual hygiene compared to girls younger than 16. Similarly, people who understood menstruation better were 1.59 (AOR: 1.59, 95% CI: 1.43, 2.94) times more likely to manage their monthly hygiene compared to people who didn’t. Compared to girls whose mothers worked for the government, teenage girls whose mothers worked for private companies were 70% (AOR: 0.30, 95% CI: 0.09, 0.99) less likely to practice good menstrual hygiene, 48% less likely (AOR: 0.52 (95% CI: (0.28, 0.96), and 47% less likely (AOR: 0.53, 95% CI: 0.29, 0.98). Moreover, adolescent girls who talked to their parents about menstruation issues were 1.62 (AOR: 1.67, 95% CI: 1.17, 2.42) times more likely to follow proper menstrual hygiene practices. (Table 6).

**Table 6:** Factors associated with menstrual hygiene management among high school and preparatory school adolescent students, Debre Markos town, Ethiopia, 2019

Variables	**Menstrual hygiene management practice**		COR (95% CI)	AOR (95% CI)
	Good (%)	Poor (%)		
**Age group**				
13–16 years	66 (40.2)	98 (59.8)	1	1
17–19 years	194 (52.9)	173 (47.1)	1.67 (1.15, 2.42)*	2.13(1.32, 3.44) **
**Residence**				
Urban	205 (49.3)	211 (50.7)	1	
Rural	55 (47.8)	60 (52.2)	1.06 (0.7, 1.6)	1.02 (0.8, 1.9)
**Educational status of the mother**				
uneducated	70(51.9)	65(48.1)	0.86(0.52, 1.27)	0.45(0.32, 1.19)
Educated	190(48.1)	205(51.9)	1	1
**Educational status of the mother**				
uneducated	95(47.7)	104(52.3)	1.08(0.76, 1.54)	1.03(0.67, 1.95)
Educated	165(49.7)	167(50.3)	1	1
**Occupational status of the father**				
Government Employee	53(42.7)	71(57.3)	1	1
Private Employee	22(48.9)	23(51.1)	0.78(0.4, 1.55)	0.57(0.2, 1.45)
Self-Employee	169(51.2)	161(48.8)	0.71(0.47,1.08)	0.58(0.14,2.18)
Farmer	16(55.2)	13(44.8)	0.607(0.27, 1.37)	0.87(0.47, 3.67)
**Occupational status of the mother**				
Government employee	23(35.4)	42(64.6)	1	1
Private employee	9(60)	6(40)	0.37(0.12, 1.16*	0.30 (0. 09, 0.99) **
Self-employee	76(50)	76(50)	0.55(0.3, 0.99)*	0.52 (0.28, 0.96) **
House wife	149(51)	143(49)	0.53(0.3, 0.92)*	0.53 (0.29, 0.98) **
**Pocket money**				
Yes	111(52.9)	99(47.1)	1	1
No	149(46.4)	172(53.6)	1.29(0.91, 1.83)*	1.307 (0.91, 1.87)
**Family monthly income**				
Below 500 birr	13(46.4)	15(53.6)	1.21(0.56, 2.61)	1.12(0.54, 2.01)
501–1500 birr	28(39.4)	43(60.6)	1.61(0.96, 2.7)	1.41(0.43, 2.76)
Above 1500 birr	183(51.1)	175(48.9)	1	1
**Family size**				
≤ 5	166(50.2)	135(49.8)	1	1
≥ 6	119 (49.2)	123(50.8)	0.96(0.68, 1.36)	0.61(0.96, 2.34)
**Knowledge towards menstrual hygiene**				
Poor knowledge	66 (40.2)	98(59.8)	1	1
Good knowledge	194(52.9)	173(47.1)	1.67(1.17, 2.42)*	1.59 (1.43, 2.94) **
**Social support**				
Poor support	118(46.8)	134(53.2)	1.22(0.74, 2.01)	1.2(0.91, 1.83)
Moderate support	99(50.5)	97(49.5)	1.05(0.63, 1.76)	1.01(0.56, 2.61)
Strong support	43(51.8)	40(48.2)	1	1
**Presence of latrine in the school**				
Yes	223(49.6)	227(50.4)	1	1
No	37(45.7)	44(54.3)	0.86(0.53, 1.38)	0.56(0.63, 2.54)
**Presence of water in the school**				
Yes	174(49.3)	179(50.7)	1	1
No	86(48.3)	90(51.7)	1.04(0.73, 1.50)	1.02(0.83, 1.98)
**Freely discussion about menstruation with parents**				
Yes	168(54.2)	142(45.8)	1.66 (1.17, 2.35)*	1.62 (1.40, 3.34) **
No	92(41.6)	129 (58.4)	1	1

** Factors significantly associated with *p*-value of < 0.05 in multi- variable analysis

* factors *p*-value < 0.2 in bi-variable and entered in to multi-variable analysis

### Challenges of menstrual hygiene management

Twelve in-depth interviews were conducted with four males—three teachers and one maternal and adolescent health officer—and eight females—two instructors, two family members of teenagers, and four students. The age range of the participants in the qualitative study was 17–54, with a mean age of 35.9. All of the participants were eighth graders or above. Six inquiries about their menstrual cycle were posed to each participant, with additional follow-up conducted as needed. The results of the in-depth interviews revealed three major themes.

### Theme 1: challenges faced by menstruating girls to manage hygienically

During their menstrual cycle, teenage girls face a range of issues, including headaches, cramping in the abdomen, anxiety about leaking, stress, and embarrassment. They experience mental anguish, anxiety, and sadness as a result of society’s concealment of the menstrual cycle. Not only do they think it’s obvious from the exterior of their attire, they also think they are in a position of contempt. To emphasize, menstruation has an impact on their social connections as well. They also find it difficult to walk and converse openly with their friends—male and female—on the day of their period.

In addition, the respondents were asked to enumerate the reasons why adolescents did not manage their menstrual hygiene appropriately. Consequently, a lack of resources and insufficient expertise were mentioned as the two main factors. Teenage girls shy away from talking about their periods and the fact that their parents don’t know if their daughter is starting to menstruate. They struggle so much, both materially and socially, to buy and use sanitary goods in public. They persistently demand payment for sanitary items. In exceptional cases, the community might even think of having intercourse at the onset of the menstrual cycle.

The qualitative investigation indicates that there are no sanitary supplies, running water, or separate classrooms for students to change into. The participants pointed out that the school does not have a restroom, hygienic materials for changing clothes, or an alternate uniform in case the uniform gets stained or seeps through. The only school that provides these facilities is the one that does not allow showering or changing classes but does have changing rooms and uniforms. The health club leader also brought up the issue of students frequently asking questions about people they are worried about and not receiving a response, in addition to the dearth of sanitary supplies in schools and the requirement for class changes.

*“When a female student is menstruating*,* she may exhibit distinct emotions. For example*,* girls were not allowed to walk freely with their friends. They experience the psychological anguish of menstruation*,* anxiety*,* despair*,* and humiliation. Because*,* in their opinion*,* they are messy*,* disrespectful*,* and it shows when they are not wearing clothes or a uniform. Weak awareness of menstrual hygiene is the root cause of poor management of menstrual hygiene. Girls who are supporting themselves or their family should be able to afford proper menstruation hygiene. In connection with that*,* girls were unable to purchase menstrual supplies”* (**45 years old**,** health club leader; female teacher)**.

*“Social relations are impacted by menstruation. It flirts with its male pals. Women require money for things like underwear*,* soap*,* and modes since menstruation is stigmatized in society and makes females feel afraid and attacked psychologically. Lack of knowledge about menstruation is the root cause of poor menstrual hygiene. During that time*,* my family was unable to permit me to complete some tasks. For example*,* I am ashamed and have not talked openly with my family about my inability to prepare food. My families are unaware of when I begin to menstruate.”***(18 years old; grade 9 female student)**.

*“There isn’t a private changing area at our school*,* and the restroom isn’t lockable. I’m not sure if the school has access to sanitary supplies to change*,* and water is completely absent.”* (**18 years old**,** grade 11; town; female student)**.

*“There is no suitable restroom*,* changing area*,* or water supply at the school. Moreover*,* there is no water in the toilets. It is not guaranteed that the modes are arranged in an expiry order. We have frequently requested assistance from the relevant bodies through formal letters*,* but we have received no answer. Any company that supplies sanitary products should guarantee that they are not expired. A large*,* spotless*,* high-quality*,* and comfortable changing room for sanitary materials should be located in the school.”* (**45 years old**,** health club leader; female teacher)**.

### Theme 2: the effect of menstruation on girls live and education

The study found that menstruation causes problems for women, which negatively affects their academic performance by making them miss school, concentrate less in class, and not study as hard. Girls who are menstruating worry about bloodstains and other menstrual symptoms, which makes them stand out from other students and even impedes their freedom of movement by creating obstacles to candid discussion and raising awareness among other students. They are consequently not focusing on their classwork in class. They also can’t eat well due of stomach discomfort brought on by despair.

*“Girls would miss school due to a strong menstrual odor*,* and when they did show up*,* they would depart before their period. They were concerned as they were unable to focus on their studies at that time.“*** (54 years old**,** male director)**.

*“During menstruation*,* girls could not learn well the full period*,* as well as they may absent from school. In the case of pain of menstruation*,* they give less attention for their learning.”* (**45 years old**,** health club leader; female teacher)**.

### Theme 3: the responsible body and solutions needed to improve poor menstrual hygiene

Most participants said that family, the individual, and the school are all involved in managing menstruation hygienically. In practical terms, the government ought to give menstrual health first priority. Raising awareness in the community and at schools, having open discussions with parents, supplying resources from pertinent bodies, and providing enough facilities at the school level were all mentioned by the majority of participants as possible solutions. The participants also recommended that the research findings give menstrual health a high priority. A few individuals conveyed their self-assurance regarding the inherent characteristics of their menstrual cycle and their capacity to champion their entitlements.

*“Menstrual hygiene is a matter that all governmental entities and society must take seriously. Families*,* gender-specific clubs*,* and both male and female students at the school should all adore famines (setnet). Families should explain to their children that menstruation is normal when they prepare meals for them. Numerous studies on teenagers have been conducted*,* yet noticeable changes have been noted. Therefore*,* the findings of this study ought to be put into practice.”* (**30 years old**,** female director)**.

“*To keep menstrual hygiene properly at student level*,* keeping personal hygiene*,* appropriate modes changing and open discussion with pears and family. “Since menstruation is physiologic*,* female students should have self-confidence about it and the school should create awareness about it.”*(**19 years old**,** grade 12; rural; female student)**.

## Discussion

This study found that 49% of the participants maintained proper menstrual hygiene. The age of the responder, whether or not they had discussed menstruation with their parents, their level of education, and whether or not their mother worked for a private company or on her own were all associated with menstrual hygiene practices.

According to this study, almost half of teenage girls managed their menstrual hygiene well. Similar results were obtained from a study carried out in Addis Ababa (51.3%) [29]. and Adama town (57%) [24]. This result, however, is more than that of a research carried out in the Wogera, Amhara region (29.8%) [23] and in Tigray region (40.8%) [14]. This can be due to the various dates the studies were carried out, the ease of access to sanitary products, the grade levels of the adolescent female participants, or any combination of these factors. This result, however, was lower than the 90.9% of an Mhallmeda study [21]. and Indonesia (64.1%) [5]. This disparity may be attributed to differences in the sociodemographic characteristics, cultural influences on education, and the degree of candor with which parents and teenagers discuss their periods. Consequently, sanitary product accessibility and education are essential interventions to reduce the high prevalence of insufficient menstrual hygiene management practices.

The present study also found that the age of the students significantly influenced menstrual hygiene practices, with those who were over seventeen years old and twice as likely as their classmates to have good practices. This investigation was consistent with one conducted in Addis Ababa [29]. and Southern India [30]. This suggests that openness and awareness of menstrual hygiene practices may rise along with the age of schoolgirls. As a result, girls in their early adolescent years require intervention.

In a similar spirit, establishing good menstrual hygiene practices (MHM) requires talking to parents about their periods. Girls were more likely to practice proper MHM than their peers if they talked to their parents about their menstruation. The qualitative study also demonstrated the impact of the girls’ non-parental communication on their menstrual hygiene routines. This study was in agreement with one conducted in Addis Ababa [29]. and Nigeria [31]. It’s probable that parents’ expertise and experience have a beneficial impact on their children’s menstrual hygiene habits. Girls and parents should make talking about menstruation a habit in order to de-stigmatize the period and accept it as a natural occurrence.

This study also discovered a significant relationship between the mother’s profession and the girl’s practice. Girls were therefore less likely to practice good menstrual hygiene than girls whose moms worked for the government whether their mothers worked for private firms, for themselves, or as housewives. This is corroborated by the qualitative study, which found that adolescents from educated families have fewer challenges than those from uneducated familiesBoth this study and a Nekemte study suggest that children of housewives are more likely to adopt moral behavior [32]. On the other hand, a Nigerian study revealed that working is a stronger protective factor against irregular menstruation than staying at home [20]. This could be because housewives, self-employed workers, and private employees have more time to assist and interact with their adolescent children than do government employees, or it could be that government employees are too busy to impart knowledge and experience to their offspring. This implies that the government employee would rather have more time to devote to tending to their teenage children and having conversations with them about menstruation-related concerns.

Furthermore, a strong correlation was found between girls’ knowledge and MHM practice, with better knowledge translating into better MHM practice. The qualitative study also demonstrated that inadequate menstrual hygiene practices are mostly caused by a lack of knowledge about menstruation and sanitary products. This investigation aligned with a Wegera study [23], Mehallmeda [21] and Addis Ababa [29]. This suggests that the respondent’s understanding of menstruation was crucial to practicing menstruation hygiene; as a result, raising awareness among early adolescents, parents, and communities is crucial.

The study also discovered that there are significant obstacles to practicing menstrual hygiene. The majority of participants listed headache and cramping in the abdomen, fear of leaking, stress and embarrassment, anxiety, depression, and psychological trauma, as well as a lack of information and resources due to the practice’s social stigma. This study and one conducted in Mehallmeda were comparable [21]., Turkish [17], urban Slum [33], Uganda [34] and India [35].

The community may consider starting of sexual intercourse when the girls start menstruation. This study was similar with a study conducted in Tigray [15]. The qualitative investigation also discovered that virtually all schools are devoid of hygienic supplies, water, and private classrooms. Again, there is no bathroom or sanitary napkins available if the menstrual blood is soiling. This study was similar with a study done in Wollo [4], and Adama [24]. Thus, improving of the school environment to be conducive for the adolescent girls during menstruation is demanded.

## Conclusion

Of the teenage girls, over half were good at maintaining their menstrual hygiene. Menstrual practice was associated with the adolescent’s age, talks with parents, knowledge of menstrual hygiene, and the mother’s kind of work. The qualitative study also showed that ignorance, revelation of monthly onset, hostile school environment, and the cultural and economical obstacles related to using sanitary products were the main reasons why teenagers did not manage their menstrual hygiene appropriately. As a result, school-based intervention programs are required that focus on the traits that have been found and increase knowledge of menstrual hygiene management techniques.

### Electronic supplementary material

Below is the link to the electronic supplementary material.

Supplementary Material 1

## Data Availability

The data and materials that used to generate this manuscript can be accessed by request of the corresponding author. `.

## Abbreviations

CI: Confidence Interval
MHM: Menstrual Hygiene Management
SD: Standard Deviation
SDG: Sustainable Development Goal
SPSS: Statistical package for Social Science
