# Supplementary material for: Menstrual hygiene management practice and associated factors among high school and preparatory school adolescent students in Debre Markos town, Northwest, Ethiopia: a mixed-method study
Source: BMC Womens Health. 2024 Jul 25;24:420. doi: 10.1186/s12905-024-03265-y (PMC11270815; doi:10.1186/s12905-024-03265-y)
Supplement: Supplementary file 1 — Supplementary Material 1 [file 12905_2024_3265_MOESM1_ESM.docx]

**Consent form**

**Consent form in English language**

**Dear study participants!**

You are invited to participate in a research project about menstrual hygiene management and associated factors among high school and preparatory adolescent students in Debre Markos town, Ethiopia.

This self administered questionnaire may take about 15 to 20 minutes to complete. Participation is voluntary, and responses will be kept anonymous.

You have the option to not respond to any questions that you choose. Participation or non participation will not affect your relationship with the teacher or any other members of the school. If you have any questions about the research, please contact the Principal Investigator,

Yichalem worku, via phone number 2519 23 52 76 71

Please put your Signature----------------------

Are you voluntary to participate in the study please?

A. No; thank you (Stop here)

B. Yes Signature -------------------------

**Data collection tool**

**Quantitative English Questionnaire**

Debre Markos University **College of Health Science Department of Midwifery for Postgraduate program**; Questionnaires for assessment of menstrual hygiene management and associated factors among high school and preparatory adolescent students.

| Code | | | | |
| --- | --- | --- | --- | --- |
| **Part 1:-Socio-demographic related questions** | | | | |
| S no | Questions | Possible answer | |  |
| 101 | How old are you now? | I am ________years | |  |
| 102 | Have you started your menses? | 1. Yes  2. No (Thank you. Please return the questionnaire before proceeding to the next questions.) | |  |
| 103 | How old were you at your menarche? | I was _______years old. | |  |
| 105 | Grade level? | 1. 9^th^ 2. 10^th^ 3. 11^th^ 4. 12^th^ | |  |
| 106 | Residence? | 1. Urban  2. Rural | |  |
| 107 | Religion? | 1. Orthodox  2. Muslim  3. Others; specify-------------- | |  |
| 108 | Ethnicity? | 1. Amhara  2. Other; specify--------------------------- | |  |
| 109 | What is your fathers’ educational status? | 1. Can’t read and write 2. Primary school 3. Secondary school 4. College level and above | |  |
| 110 | What is your mothers’ educational status? | 1. Can’t read and write 2. Primary school 3. Secondary school 4. College level and above | |  |
| 111 | What is the occupational status of your father? | 1. Government Employee  2. Private Employee  3. Self-Employee  4. Farmer  5. Others; specify -------------- | |  |
| 112 | What is the occupational status of your mother? | 1. Government Employee 2. Private Employee 3. Self-Employee 4. House wife 5. Other; specify----------------- | |  |
| 113 | How much does your family earn per month on average? | My family earns----------ETB on average/month | |  |
| 114 | How many members are there in your family? | _____________ | |  |
| 115 | Do your parents provide regular pocket money regularly for you? | 1. Yes  2. No | |  |
| **Part 2:- Knowledge related questions.** | | | | |
| 201 | Have you heard about menstruation before menarche? | 1. Yes  2. No | |  |
| 202 | If your answer is ‘Yes’ for question no “**201”,** where did you get the information about menstruation before menarche?  ( more than one option is possible) | 1. Mother.  2. Teacher  3. Peers.  4. mass media  5. Others; specify------------- | | If your answer is “no” skip to Q 203 |
| 203 | At what age, do you think a healthy girl usually get her first menses? | __________years old | |  |
| 204 | What do you think about the cause of menstruation? | 1. Physiological process.  2. Is caused by a sin.  3. Is curse of God?  4. Is caused by a disease.  5. I don‘t know. | |  |
| 205 | From which organ does the menstrual blood come? | 1. Vagina  2. Urinary bladder  3. Uterus  4. I don‘t know | |  |
| 206 | How long is the normal menstrual bleeding duration? | 1. <2 Days.  2. 2-7 Days  3. >7 Days  4. Don’t know | |  |
| 207 | What is the normal duration of menstrual cycle? | 1. <20 Days  2. 20-35 Days  3. >35 Days  4. Don’t know | |  |
| 208 | Do you think menstruation is a secret issue? | 1. Yes  2. No | |  |
| 209 | If your answer is “yes” for question no “**208”**, why? (more than one option is possible) | 1. Deep rooted culture of the society.  2. Believes and customs of the society  3. Others; specify---------------------------- | | If your answer is “no” skip to Q“210” |
| 210 | Do you know sanitary pads in the market? | 1. Yes  2. No | |  |
| 211 | Can poor menstrual hygiene predispose to infection? | 1. Yes  2. No | |  |
| 212 | Does menstrual hygiene have a contribution in prevention of menstrual pain? | 1. Yes  2. No | |  |
|  |  |  | |  |
| **Part 3. Menstrual hygiene management related questions** | | | |  |
| 301 | Do you use sanitary material(s) during menstruation? | | 1. Yes  2. No |  |
| 302 | If your answer is “Yes” for question no “**301”**, what sanitary material do you use during menstruation? | | 1. Disposable sanitary pads.  2. Disposable piece of rags.  3. Reusable sanitary pads  4. Underwear.  5. Others; specify------------------------- | If your answer is “no” skip to Q“303” |
| 303 | If your answer is “No” for question no “**301”**, why? (more than one option is possible) | | 1. Lack of knowledge  2. High cost  3. Unavailability  4. Shyness  5. Others ; specify------------------------ | If your answer is “yes” skip to Q “304” |
| 304 | Do you wash your genitalia during menstruation? | | 1. Yes  2. No |  |
| 305 | If your answer for question no “**304”** is “yes*”* what medium do you use for your genital cleaning purpose? | | 1. Only Water.  2. Soap and water | If your answer is “no” skip to Q“307” |
| 306 | If your answer for question no “**304”** is “yes*”* how often doing you wash your genitalia per day? | | ------------------------- |  |
| 307 | Do you take bath during menstruation (exceptional from the usual)? | | 1. Yes  2. No |  |
| 308 | Do you change your sanitary material(s) during menstruation at school? | | 1. Yes  2. No |  |
| 309 | If your answer for question no “**308”** is “yes*”* how often do you change your sanitary material (s) during menstruation per day? | | _____________ | If your answer is “no” skip to Q“310” |
| 310 | How do you dispose of menstrual materials after use? | | 1. Open field  2. Latrine  3. Wrap in paper and put in the bin  4. Others; specify --------------------- |  |
| 311 | Where do you store your new and/or reusable absorbent(s)? | | 1. Drawers  2. Dress cabinet  3. Bathrooms.  4. Store with routine cloth.  5. Others; specify------------------------- |  |
| 312 | Where do you put/keep your reusable sanitary pads after washing for drying? | | 1. In the sunlight outside  2. In the shade inside  3. others___________ |  |
| 313 | Is the school is comfortable to keep hygiene during menstruation? | | 1. Yes 2. No |  |
| **Part 4. Environmental related questions** | | | | |
| 401 | Does the school have functional water source? | | 1. Yes 2. No |  |
| 402 | Does the school have functional toilet facility? | | 1. Yes 2. No |  |
| 403 | Are females and males toilets in the opposite directions? | | 1. Yes 2. No |  |
| 404 | Are females’ toilets kept locked inside? | | 1. Yes 2. No |  |
| 405 | Is your school giving guidance and counseling service on menstrual hygiene management? | | 1. Yes 2. No |  |
| 406 | Is there any organization who gives sanitary materials in your school? | | 1. Yes 2. No |  |
| **Part 5. psycho social related questions** | | | | |
| 501 | Do you freely discuss about menstruation issues with your parents? | | 1. Yes  2. No |  |
| 502 | If your answer for question no **501** is “Yes”, in what topics/issues why?  (more than one option is possible) | | 1. about menstrual hygiene management.  2. About methods how to use sanitary pads.  3. others ------------ | If your answer is “no” skip to Q“503” |
| 503 | If your answer for question no **501** is “No”, why? (more than one option is possible) | | 1. Because of shamefulness  2. Not usual.  3. Privacy.  4. Other (specify)______ | If your answer is “yes” skip to Q 504 |
| 504 | What do you feel in the class during menstruation? (more than one option is possible) | | 1. Discomfort 2. Unable to give attention 3. Fear to stand in front of class 4. Other specify_________ |  |
| 505 | Have you absented from school during your menses in the resent or the previous month? | | 1. Yes 2. No |  |
| 506 | If your answer is “yes” for question no **505** why? (more than one option is possible) | | 1. Pain 2. Fear, shame, leakage or stain, lack of confidence 3. Lack of disposal 4. Lack of water to wash 5. Lack of privacy to change 6. Lack of sanitary material 7. Lack of separate bathroom | If your answer is “no” skip to Q“507” |
| 507 | If your answer is “yes” for question no **505** how many days per cycle? | | ______ days/cycle |  |
| 508 | If your answer is “1” for question no 506’  What is your solution? (more than one option is possible) | | 1. Visit clinic 2. Anti-pain from pharmacy 3. Homemade remedy 4. Nothing | If your answer is “not 1” skip to Q “509” |
| 509 | Who is your intimate guidance related to menstruation? (more than one option is possible) | | 1. Mother 2. Father 3. Sister 4. Female peer 5. Male peer 6. Others specify________ |  |

| **Part 6. The Oslo 3-items social support scale** | | |
| --- | --- | --- |
| 601 | How easy can you get help from neighbours if you should need it? | 1. very difficult 2. difficult 3. possible 4. easy 5. Very easy |
| 602 | How many people are so close to you that you can count on them if you have serious problems? | 1. None 2. 1-2 3. 3-5 4. 5+ |
| 603 | How much concern do people show in what you are doing? | 1. No 2. Little 3. Uncertain 4. Some 5. A lot |

**Qualitative In-depth interview Guide**

**Part one. Socio-demographic characteristics**

1. Code____________
2. Age ______________
3. Sex__________________
4. Residence_________________
5. Level of education_____________
6. Occupation _______________
7. Experience in the current position ___________

**Part two. IDI Checklist for menstrual hygiene management (for all key-informants)**

1. What do you think about the problems that female students face during menstruation? (Probe social, environmental, and economical problems)
2. What are the causes of poor menstrual hygiene management among adolescent girls? (Probe: knowledge, family related factors, school environmental related factors, cultural reasons, …)
3. What are the effects of menstruation on the girls’ academic performance?

(Probe health effects, school absenteeism, and dropout, psychological effects, ---)

1. What looks like the sanitation condition of the school to manage menstruation hygienically at school?

(Probe water, private toilet, any sanitary material supporter ---)

1. Who is responsible, do you think for menstrual hygiene management? (probe:
2. What solution do you suggest for proper menstrual hygiene management (PROBE: at individual level (student level), family level, school level, gender clubs, etc…)
3. Is there anything you would like to add that we didn’t discuss above?
